# Genomics is modifying our concepts of evolution in homosporous and heterosporous vascular plants

**DOI:** 10.1002/ajb2.70248

**Published:** 2026-08-11

**Authors:** Sylvia P. Kinosian

**Affiliations:** ^1^ Department of Plant Sciences and Plant Pathology Montana State University Bozeman 59715 Montana USA

**Keywords:** chromosome structure, genome biology, polyploidy, reproductive mode

## Abstract

Across the tree of life, and particularly within land plants, there is a wide diversity and disparity of genome structure. The most well‐studied are angiosperms, comprising mostly small and dynamic genomes, with a history of ancient whole genome duplications. Genome structure and evolution have historically been less clear in ferns and lycophytes, and the origin of their large genomes and high chromosome numbers has been debated for decades, centering on differences in reproductive mode (heterospory and homospory). Klekowski (1973) hypothesized that homosporous ferns and lycophytes were mainly inbreeding and that polyploidy allowed for higher genetic diversity via homoeologous heterozygosity. In contrast, Haufler (1987) and others proposed that homosporous ferns and lycophytes had diploid inheritance and outcrossed frequently, a theory supported by genetic crossing, isozymes, and modern comparative genomic analyses. This article summarizes past and current theories on the mechanisms of genome evolution in homosporous plants, highlights the contributions of comparative genomics to this field in the past two decades, and discusses what we may be able to learn by considering the role of meiosis in heterosporous and homosporous fern and lycophyte genome structure.

Genome size and structure are incredibly varied across the tree of life and particularly within land plants (e.g., Kress et al., 2022). The most well‐studied are angiosperms, consisting of diverse genomes with dynamic evolutionary histories and frequent whole genome duplications (WGDs; e.g., Soltis et al., 2008; Tiley and Burleigh, 2015; Wendel, 2015; One Thousand Plant Transcriptomes Initiative, 2019; Pellicer and Leitch, 2020; Wang et al., 2021; Almeida‐Silva and Van de Peer, 2023; Bowles and Paps, 2025 [preprint]). Gymnosperm genomes typically are large, have fewer WGDs than angiosperms, and are characterized by large proportions of repeat elements (Leitch and Leitch, 2013; Wan et al., 2022). Bryophytes generally have small genomes, variable frequencies of WGDs, and disparate gene organization compared to seed plants (Lang et al., 2018; Szövényi et al., 2021; Dong et al., 2025; Fujiwara et al., 2025; Patel et al., 2025; Schafran et al., 2025). Ferns and lycophytes (a paraphyletic group of spore‐dispersed vascular plants) tend to have large genomes and high chromosome numbers compared to other land plants, but the evolution of their genome structure has been debated for decades (e.g., Klekowski and Baker, 1966; Haufler and Soltis, 1986; Haufler, 1987; Soltis and Soltis, 1987; Nakazato et al., 2008; Barker, 2013; Kinosian et al., 2022; Dhakal et al., 2025).

Many theories of fern and lycophyte genome evolution converge on a correlation of reproductive mode (heterospory vs. homospory) with genome structure: heterosporous plants typically have small genomes and low chromosome numbers, while homosporous plants often have large genomes and high chromosome numbers (Table 1; Clark et al., 2016; Szövényi et al., 2021; Kinosian et al., 2022; Plačková et al., 2024). Homospory is the ancestral character state in land plants: one type of spore germinates into a gametophyte capable of producing eggs, sperm, or both (Figure 1A). All extant bryophytes, most ferns and lycophytes, and the extinct ancestors of seed plants are homosporous. Heterospory is a derived character state in land plants: separate megaspores and microspores develop into egg‐ and sperm‐producing gametophytes, respectively (Figure 1B). There are three extant lineages of heterosporous plants, including two orders of lycophytes, one order of ferns, and all seed plants. Importantly, these lineages represent independent evolutions of heterospory (Bateman and DiMichele, 1994), and all share similar genome traits (Szövényi et al., 2021; Kinosian et al., 2022). Ferns and lycophytes each have an independent evolution of heterospory alongside extant homosporous relatives, creating a unique study system to investigate the role of reproductive mode on genome structure. There are fundamental differences in meiosis between heterosporous and homosporous plants, which may be an important aspect of post‐WGD evolution in different lineages of land plants (Plačková et al., 2024; Kinosian and Barker, 2025). Decades of work on fern and lycophyte genome evolution have laid the foundation for modern comparative genomics, which is quickly advancing our understanding of genome evolution in heterosporous and homosporous vascular plants.

**Table 1:** Published genome sequences for heterosporous and homosporous fern and lycophyte species. Species are ordered (1) by major group (ferns then lycophytes), (2) by reproductive mode (heterosporous then homosporous), and (3) alphabetically within reproductive mode. Families are derived from PPG (2016).

Species	Family	Reproductive mode	Genome size (1C, GB)	Chromosome number (n)	Citation
*Azolla caroliniana*	Salviniaceae	Heterosporous	0.521	22	Song et al. (2025)
*Azolla filiculoides*	Salviniaceae	Heterosporous	0.75	22	Li et al. (2018)
*Marsilea vestita*	Marsileaceae	Heterosporous	1.34	20	Rahmatpour et al. (2023)
*Salvinia cuculata*	Salviniaceae	Heterosporous	0.25	9	Li et al. (2018)
*Isoetes sinensis*	Isoetaceae	Heterosporous	2.13	22	Cui et al. (2022)
*Isoetes taiwanenesis*	Isoetaceae	Heterosporous	1.66	11	Wickell et al. (2021)
*Selaginella kraussiana*	Selaginellaceae	Heterosporous	0.132	10	Liu et al. (2023)
*Selaginella lepidophylla*	Selaginellaceae	Heterosporous	0.109	10	VanBuren et al. (2018)
*Selaginella moellendorffii*	Selaginellaceae	Heterosporous	0.102	10	Banks et al. (2011); Xiong et al. (2025)
*Selaginella tamariscina*	Selaginellaceae	Heterosporous	0.301	10	Xu et al. (2018)
*Adiantum capillus‐venerus*	Pteridaceae	Homosporous	4.8	30	Fang et al. (2021)
*Adiantum nelumboides*	Pteridaceae	Homosporous	7.39	60	Zhong et al. (2022)
*Alsophila spinulosa*	Cyatheaceae	Homosporous	6.23	69	Huang et al. (2022)
*Ceratopteris richardii*	Pteridaceae	Homosporous	9.6	39	Marchant et al. (2019), (2022)
*Cibotium barometz*	Cibotiaceae	Homosporous	3.47	66	Qin et al. (2024)
*Dipteris shenzhenensis*	Dipteridaceae	Homosporous	1.9	33	Shu et al. (2025)
*Dryopteris fragrans*	Dryopteridaceae	Homosporous	4.45	41	Chang et al. (2026) [preprint]
*Gymnosphera denticulata*	Cyatheaceae	Homosporous	6.25	68	Wei et al. (2025)
*Lygodium microphyllum*	Lygodiaceae	Homosporous	4.75	30	Pelosi et al. (2025)
*Sphaeropteris brunoniana*	Cyatheaceae	Homosporous	2.54	69	Wei et al. (2025)
*Sphaeropteris lepifera*	Cyatheaceae	Homosporous	5.50	69	Wei et al. (2025)
*Diphasiastrum complanatum*	Lycopodiaceae	Homosporous	1.7	24	C. Li et al. (2024)
*Huperzia asiatica*	Lycopodiaceae	Homosporous	7.8	69	C. Li et al. (2024)
*Lycopodium clavatum*	Lycopodiaceae	Homosporous	2.3	34	Yu et al. (2023)

**Figure 1:**
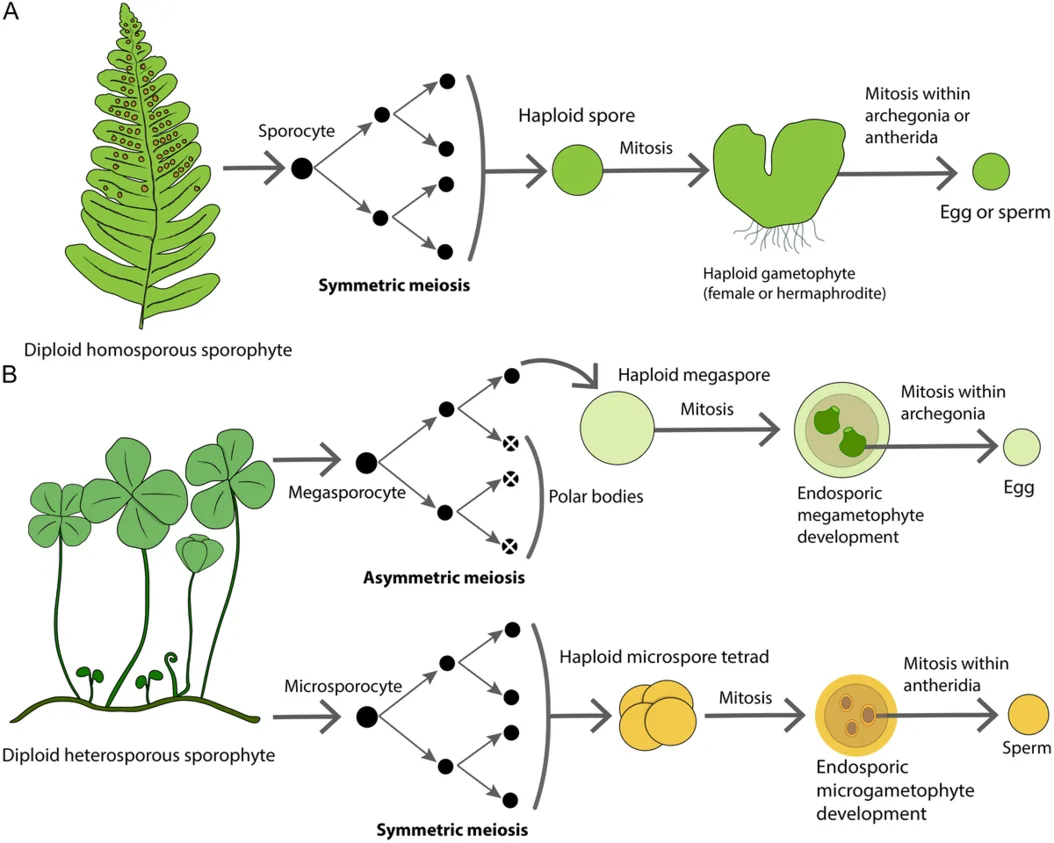
Sporogenesis and gametogenesis of (A) homosporous and (B) heterosporous vascular plants. Homosporous sporogenesis can proceed as depicted, with a sporocyte going through one round of meiosis to produce four haploid spores; this is the case for homosporous lycophytes and eusporangiate ferns. In leptosporangiate ferns, the sporocyte goes through four rounds of mitosis and then a round of meiosis to produce 64 haploid spores.

Christopher Haufler categorized three periods of significant discovery in fern and lycophyte genomics (Haufler, 2014): (1) work on fern and lycophyte breeding systems and inheritance patterns by Irma Andersson‐Kottö (Andersson, 1927; Andersson‐Kottö, 1929, 1938), (2) the extensive contributions to fern and lycophyte cytology by Irene Manton (e.g., Manton, 1950), and (3) Edward Klekowski's hypotheses on fern and lycophyte breeding systems and chromosome numbers (e.g., Klekowski and Baker, 1966; Klekowski, 1973). I propose the addition of two further periods in this series, both driven by efforts from a community of scientists: (4) the discovery that ferns and lycophytes with high chromosome numbers are diploid and outcrossing (reviewed in Soltis and Soltis, 1987; Barker, 2009; Haufler, 1987, 2002, 2014) and (5) the characterization of structural variation and evolutionary mechanisms of fern and lycophyte genomes (e.g., Marchant et al., 2022; Pelosi et al., 2022; Zhong et al., 2022; Wei et al., 2025). Here, I review the work being done in this fifth period, including studies on the frequency and consequences of WGD (e.g., Barker, 2009; Wolf et al., 2015; Chen et al., 2022; Pelosi et al., 2022; Z. Li et al., 2024 [preprint]), genome structure and chromosome number (e.g., Liu et al., 2019; Fujiwara et al., 2021; Z. Li et al., 2024 [preprint]), comparative genomic analyses (e.g., Pelosi et al., 2022; Fernández et al., 2023), and published genomes for heterosporous and homosporous ferns and lycophytes (Table 1). Synthesizing these findings, I discuss how we might incorporate meiosis dynamics into the broader framework of fern and lycophyte genome evolution. After years of fern and lycophyte genomics lagging behind research in other plant groups (Barker and Wolf, 2010; Sessa et al., 2014; Szövényi et al., 2021), we now have the resources to characterize not only the broad patterns of their genome structure and evolution, but the nuances and unique evolutionary mechanisms found among their component lineages.

## HISTORICAL CONTEXT

Research on fern and lycophyte genomics is nearing its centennial. In the 1920s and 1930s, Andersson‐Kottö established that ferns have disomic and Mendelian inheritance, as confirmed by genetic crossing experiments (Andersson, 1927; Andersson‐Kottö, 1929, 1938). Our understanding of fern and lycophyte genome and chromosome structure began soon after with the pioneering cytogenomic work of Manton (Manton 1939, 1950; Manton and Sledge, 1954; Tryon and Britton, 1958; Manton and Vida, 1968). Klekowski and colleagues took the next step in fern and lycophyte genomics, with their observation of chromosome number disparity between homosporous and heterosporous vascular plants (Klekowski and Baker, 1966). To reconcile the correlation between reproductive mode and chromosome number, Klekowski (1973) hypothesized that homosporous ferns and lycophytes with high chromosome numbers were polyploid and primarily inbreeding. Specifically, the homoeologous heterozygosity in polyploids was predicted to enable purging of genetic load built up by intragametophytic selfing (also known as gametophytic selfing; Haufler et al., 2016). This type of extreme selfing is unique to homosporous plants, in which a bisexual gametophyte produces genetically identical sperm and egg cells, which fuse to create a completely homozygous zygote (see Haufler et al., 2016: Figure 1).

Klekowski's hypothesis was instrumental in driving research on fern cytology and reproductive biology throughout the mid‐20th century. However, work on fern breeding systems with isozymes reconfirmed the predictions of Andersson‐Kottö that ferns are primarily outcrossing and have disomic, not multisomic, inheritance (Gastony and Gottlieb, 1985; Haufler and Soltis, 1984, 1986; Wolf et al., 1987; Soltis and Soltis 1987, 1992). These findings disproved Klekowski's hypothesis and suggested that differences in genome structure and mechanisms of genomic downsizing following WGD may be the root cause of the disparate chromosome numbers between heterosporous and homosporous vascular plants (Haufler, 1987; Soltis and Soltis, 1987; Gastony, 1991).

**Figure 2:**
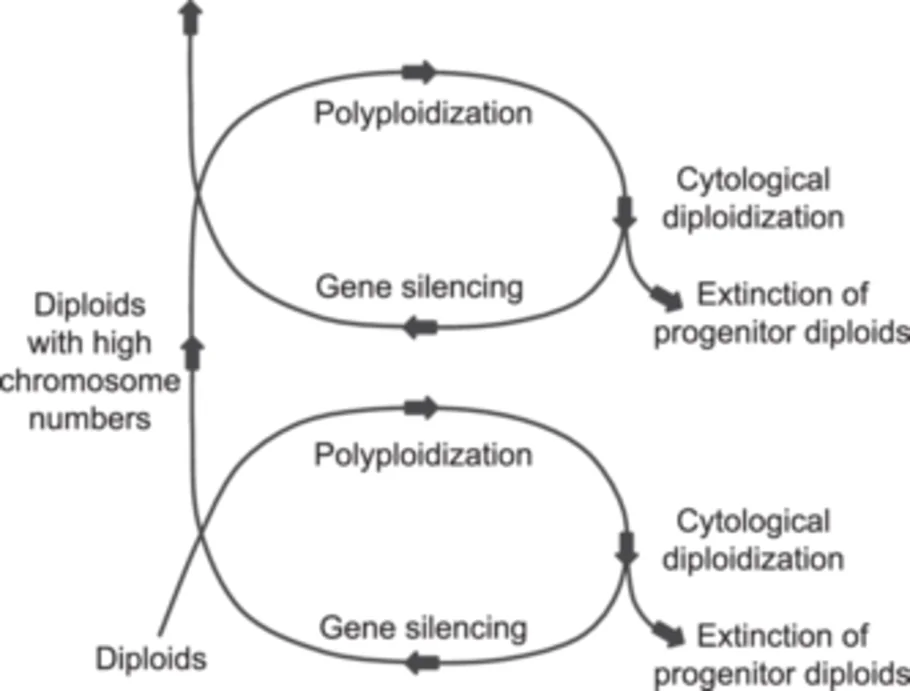
The upward spiral hypothesis of homosporous fern and lycophyte genome evolution (adapted from Haufler, 1987).

In accordance with these new data, Haufler (1987) proposed that homosporous ferns and lycophytes follow an upward spiral of genome size and chromosome number, due to a lack of genome downsizing following polyploidy (Figure 2). Following a whole genome duplication, homosporous ferns and lycophytes quickly return to disomic inheritance and silence—but do not delete—duplicated genes (Haufler, 1987). Assuming several cycles of polyploidy, this would result in large genomes and high, polyploid‐like chromosome numbers and diploid inheritance (Haufler, 1987). Heterosporous angiosperms, in contrast, rapidly downsize both genome size and chromosome number following whole genome duplication (Wendel, 2015). Haufler's theory provided a working hypothesis to explain the disparate genome structures and evolutionary mechanisms between homosporous and heterosporous vascular plants.

## CHARACTERIZING STRUCTURAL VARIATION AND THE ROLE OF MEIOSIS IN FERN AND LYCOPHYTE GENOMES

In the nearly forty years since its proposal, most aspects of the upward spiral hypothesis (Haufler, 1987) have been tested with a combination of isozyme and genomic data, including the contributions of cycles of WGD, chromosome number variation, and mechanisms of diploidization to genomic structure. More recently, transcriptome and nuclear genome assemblies have been published for heterosporous and homosporous ferns and lycophytes, enabling comparative genomic work at a larger scale than was previously possible. Below, I review some of the advances made in our understanding of evolutionary mechanisms, chromosome structural variation, and the role of meiosis in fern and lycophyte genome evolution.

The upward spiral hypothesis posits that the high chromosome numbers and large genomes of homosporous vascular plants are a result of many rounds of WGD in comparison to heterosporous angiosperms (Haufler, 1987). An early analysis of WGDs in ferns and lycophytes determined that neither lineage has a high number of WGDs in its evolutionary history (Nakazato et al., 2008; Barker, 2009, 2012). Recent work using transcriptomes showed that heterosporous and homosporous ferns and lycophytes have roughly the same number of WGDs in their evolutionary history as angiosperms, suggesting that differences in diploidization mechanisms could explain the disparate genome structures between reproductive modes (One Thousand Plant Transcriptomes Initiative, 2019; Huang et al., 2020; Chen et al., 2022; Pelosi et al., 2022; Z. Li et al., 2024 [preprint]).

There are two main mechanisms of post‐WGD diploidization in plants: cytological and genic diploidization. Cytological diploidization is the process by which the multisomic inheritance of neopolyploids is returned to the disomic inheritance of diploids via large‐scale chromosomal rearrangements (Ramsey and Schemske, 2002; Ma and Gustafson, 2005). Genic diploidization is the extensive gene silencing and loss that polyploid genomes undergo following WGD (Ma and Gustafson, 2005; Schnable et al., 2011; Mandáková and Lysak, 2018). In cytological diploidization, a continuum of multivalent to bivalent pairing is expected to happen as neopolyploids transition toward diploidy (Le Comber et al., 2010; Li et al., 2021). However, many neopolyploid homosporous ferns have strong bivalent pairing (e.g., Manton, 1950; Vida, 1970; Wagner and Wagner, 1980; Grusz et al., 2021), which suggests that cytological diploidization may occur quickly and/or via mechanisms other than extensive chromosomal changes (Huang et al., 2022; C. Li et al., 2024). Cytological diploidization with limited chromosomal rearrangements could be an explanation for the remarkably conserved intergenomic synteny found between some genomes of homosporous ferns and lycophytes (Huang et al., 2022; C. Li et al., 2024). However, other homosporous vascular plant genomes show much more rapid cytological diploidization paired with chromosomal rearrangements (Marchant et al., 2022; Wei et al., 2025), so this must be investigated carefully among lineages.

Genic diploidization in angiosperms is thought to occur through the biased deletion of genes from one genome in a polyploid (Wendel, 2015). Comparatively, in homosporous ferns and lycophytes, genic diploidization may happen via gene silencing (pseudogenization), rather than gene deletion (Haufler, 1987; Barker, 2013; Li et al., 2021). Isozyme work from the 1990s on three different homosporous ferns led to the discovery of silenced copies of several genes (Pichersky et al., 1990; Gastony, 1991; McGrath et al., 1994). Many silenced genes were detected in the linkage map for *Ceratopteris richardii*, but extensive chromosomal rearrangements meant that duplicated genes could not be compared across homoeologous chromosomes (Nakazato et al., 2006). More recently, a large number of pseudogenes were found in the tetraploid homosporous fern *Adiantum nelumboides* (Zhong et al., 2022). In addition, an analysis of paralogs in ferns and lycophytes found no significant change in the proportion of retained paralogs through time, suggesting that genes are not deleted as quickly following WGD as in angiosperms (Li et al., 2021). These insights support that genetic diploidization proceeds by gene silencing rather than gene deletion in homosporous ferns and lycophytes. However, we currently lack comparative work on how genic diploidization proceeds in these groups, which is necessary to fully understand genome diploidization and downsizing in homosporous vascular plants.

In addition to the processes of WGD and diploidization, chromosome size and structural evolution has been considered as a way to explore the genomic disparities between heterosporous and homosporous vascular plants. We know that, on average, genomes of homosporous vascular plants have a large number of small, uniform chromosomes compared to heterosporous angiosperms (Wagner and Wagner, 1980; Nakazato et al., 2008; Clark et al., 2016; Fujiwara et al., 2021) and are the only lineages with a positive correlation between chromosome number and genome size (Nakazato et al., 2008). These observations, coupled with evidence that high chromosome numbers are ancestral in homosporous ferns and lycophytes (Clark et al., 2016), have led some researchers to suggest that homosporous fern and lycophyte chromosomes do not experience substantial structural variation (Nakazato, 2009). However, some homosporous fern and lycophyte groups do not show a correlation between chromosome number and genome size (Bainard et al., 2011; Clark et al., 2016; Liu et al., 2019) and have variable chromosome sizes (Dyer et al., 2013; Liu et al., 2019; Fujiwara et al., 2021). How fern and lycophyte chromosome size and structure evolve is not clear from current data, including the role of recombination rate and amount of repetitive elements. The model fern *C. richardii* has fewer recombination events per chromosome than angiosperms (Nakazato et al., 2006, 2008). Recombination can be higher in gene‐rich regions (Cai and Xu, 2007), so given the low gene density of ferns (Rabinowicz et al., 2005; Szövényi et al., 2021; Wickell et al., 2021; C. Li et al., 2024), it could be possible that they have relatively low rates of recombination. It is notable that homosporous ferns and lycophytes have a larger proportion of repeat elements than heterosporous ferns, lycophytes, or angiosperms (Baniaga and Barker, 2019; Marchant et al., 2019, 2022; Fang et al., 2021; Wickell et al., 2021; Yu et al., 2023; C. Li et al., 2024). If major chromosomal changes are uncommon in homosporous fern and lycophyte genomes, genome restructuring on a smaller spatial scale could be a mechanism of post‐WGD evolution (Wei et al., 2025) and an explanation of low rates of gene loss and high chromosomal stability (Cai and Xu, 2007).

An alternative hypothesis regarding homosporous fern and lycophyte chromosome structural variation considers the role of the extreme self‐fertilization (known as intragametophytic or gametophytic selfing; Haufler et al., 2016) possible in homosporous vascular plants. Marchant (2019) proposed that intragametophytic selfing in homosporous plants could provide a mechanism to restore meiotic stability in cases of major chromosomal abnormalities. Intragametophytic selfing creates a completely homozygous individual in one generation, and so, regardless of structural changes from the previous generation, each chromosome would have an identical counterpart, enabling bivalent pairing (Marchant, 2019). Estimates show that there is widespread, but not comprehensive, capacity for gametophytic selfing across homosporous ferns and lycophytes (Soltis and Soltis, 1992; Sessa et al., 2016). However, even if infrequent, such a breeding system could help a lineage recover from chromosomal changes (such as WGD or rearrangements) that would otherwise create sterile offspring due to pairing issues during meiosis (Marchant, 2019).

A final potential mechanism for the differences in genome structure between homosporous and heterosporous plants is the symmetry of meiosis. Heterosporous female meiosis is asymmetric, meaning that only one of four daughter cells survives during meiosis and the remaining three polar bodies die (Figure 1B). Heterosporous male and all homosporous meiosis is symmetric, whereby all daughter cells survive (Figure 1A,B). An important caveat is that female meiosis in the heterosporous lycophyte *Isoetes* is symmetric (Smith, 1900; Pettitt, 1971; Bell, 1985) and that the number of surviving daughter cells is variable in *Selaginella*; however, more work on megasporogensis in *Selaginella* is needed given the observed variability in the number of surviving megaspores (Campbell, 1930; Pettitt, 1971, 1977; Gifford and Foster, 1989). Interestingly, both these genera have similar chromosome numbers to other heterosporous plants, but *Isoetes* has larger genome sizes (Table 1). The asymmetry of female heterosporous meiosis can lead to rapid genomic changes through a type of nonrandom chromosome segregation during meiosis called female meiotic drive (Pardo‐Manuel de Villena and Sapienza, 2001; Lindholm et al., 2016; Plačková et al., 2024; Presgraves et al., 2026). Female meiotic drive may favor chromosome fusions (Stewart et al., 2019 [preprint]; Clark and Akera, 2021) or fissions (Chmátal et al., 2014; Boman et al., 2024), depending on centromere size and strength (e.g., Clark and Akera, 2021; Plačková et al., 2024) or the polarity of drive (direction of spindle asymmetry in meiosis; see Pardo‐Manuel de Villena and Sapienza, 2001: Figure 2). Biased transmission of chromosome fusions or fissions in heterosporous plants could be part of how they achieve rapid downsizing of genome and chromosome number post‐WGD (Kinosian and Barker, 2025; Presgraves et al., 2026). In theory, female meiotic drive is not possible in organisms with symmetric meiosis (Zedek and Bureš, 2016), although other types of transmission ratio distortion may be possible (Lindholm et al., 2016). Given an absence of drive, we could expect to see relatively uniform chromosome and centromere size and structure across homosporous plants. Homosporous ferns have more uniform chromosome size than angiosperms (Dyer et al., 2013; Liu et al., 2019; Fujiwara et al., 2021). However, little is known about centromere size and structure in homosporous plants outside of hornworts (Szövényi et al., 2021; Schafran et al., 2025). Dedicated sequencing efforts are needed to assemble homosporous and heterosporous fern and lycophyte centromeres, because characterizing their structural variation will help inform us how kinetochore bonding, bivalent pairing, and chromosome segregation occur during meiosis in lineages with symmetric and asymmetric meiosis. Overall, the lack of female meiotic drive as a mechanism of genomic change in homosporous vascular plants could help explain their slower rates of dysploidy (Z. Li et al., 2024 [preprint]) and relative karyotypic stasis (Nakazato, 2009; Clark et al., 2016) compared to their heterosporous relatives.

## CONCLUSIONS

To date, in the fifth major period of research on fern and lycophyte genomics, we have accrued evidence that homosporous ferns and lycophytes have large genomes and high chromosome numbers compared to heterosporous plants due to limited chromosomal rearrangements or gene loss following WGD (Li et al., 2021; Zhong et al., 2022; C. Li et al., 2024; Z. Li et al., 2024 [preprint]), but there is growing evidence that considerable variation exists in post‐WGD genome evolution across lineages (e.g., Marchant et al., 2022; C. Li et al., 2024; Wei et al., 2025). As we work to understand the genomics of ferns and lycophytes, incorporating the foundational work on their life history, inheritance, and cytology is important for building a comprehensive research framework. Investigating the genetic mechanisms of bivalent pairing in homosporous plants will be critical for understanding cytological diploidization (Haufler, 1987). Chromosome painting in neopolyploid ferns and lycophytes would provide information on how and when homologous chromosomes pair, as well as the physical location of recombination and rearrangements (e.g., Comai et al., 2003). We can also consider centromere structure in homosporous ferns and lycophytes and investigate the potential effects of non‐meiotic transmission ratio distortion on homosporous vascular plant genomes (Kinosian and Barker, 2025). Finally, developing resources for closely related species will allow for comparative work on lineage‐specific patterns of genome structural evolution (Dong et al., 2025; Schafran et al., 2025; Wei et al., 2025).

## AUTHOR CONTRIBUTIONS

S.P.K.: Conceptualization; Visualization; Writing—original draft; Writing—review and editing.

## Data Availability

No data were generated in the preparation of this manuscript.
